# Pre-clinical carotid atherosclerosis and sCD163 among virally suppressed HIV patients in Botswana compared with uninfected controls

**DOI:** 10.1371/journal.pone.0179994

**Published:** 2017-06-29

**Authors:** Mosepele Mosepele, Linda C. Hemphill, Walter Moloi, Sikhulile Moyo, Isaac Nkele, Joseph Makhema, Kara Bennett, Virginia A. Triant, Shahin Lockman

**Affiliations:** 1 Department of Medicine, Faculty of Medicine, University of Botswana, Gaborone, Botswana; 2 Botswana-Harvard AIDS Institute Partnership, Gaborone, Botswana; 3 Harvard Medical School & Massachusetts General Hospital (Division of Cardiology-LCH and Divisions of Infectious Diseases and General Internal Medicine-VAT), Boston, Massachusetts, United States of America; 4 Bennett Statistical Consulting Inc, Ballston Lake, New York, United States of America; 5 Department of Immunology & Infectious Diseases Harvard T.H. Chan School of Public Health, Boston, Massachusetts, United States of America; 6 Division of Infectious Diseases, Brigham & Women`s Hospital, Boston, Massachusetts, United States of America; Azienda Ospedaliera Universitaria di Perugia, ITALY

## Abstract

**Objectives:**

Human immune deficiency virus (HIV) is associated with increased cardiovascular disease (CVD) risk, yet the relationship between HIV and carotid atherosclerosis / monocyte activation among virally suppressed HIV-infected patients in sub-Saharan Africa is not well understood.

**Methods:**

We measured traditional CVD risk factors, bilateral distal common carotid intima media thickness (cIMT), presence of carotid plaque and plasma sCD163 levels among virally suppressed HIV-infected adults and HIV-uninfected controls, in a cross-sectional study in Gaborone, Botswana. The associations between HIV status, traditional CVD risk factors, sCD163 and outcome of cIMT were assessed in univariate and multivariate linear regression models.

**Results:**

We enrolled 208 HIV-infected adults (55% Female, mean age 39 years) who had undetectable HIV-1 RNA on antiretroviral therapy and 224 HIV-uninfected controls (47% Female, mean age 37 years). There was no difference in cIMT between study groups, with mean cIMT 0.607mm and 0.599mm in HIV-infected and HIV-uninfected, respectively (p = 0.37). Plasma sCD163 was significantly higher in HIV-infected versus HIV-uninfected persons (1917ng/ml vs 1593ng/ml, p = 0.003), but was not associated with cIMT (p = 0.43 among all, p = 0.72 for HIV-infected only). In the final multivariate model, increased cIMT was associated with older age, being treated for hypertension, and higher non-HDL cholesterol among all (p<0.001, p = 0.03, p<0.001 respectively), and with older age and waist-hip ratio in HIV-infected participants (p = 0.02 & p = 0.02 respectively). Carotid plaque was present in a significantly higher proportion of HIV-infected adults (RR 2.15, 95% CI 1.22, 3.81).

**Conclusions:**

HIV-infected participants aged 30–50 years who have achieved viral suppression did not have increased cIMT when compared to HIV-uninfected controls in Botswana. However, well-controlled HIV was associated with excess monocyte activation. Future work should explore the impact of subclinical atherosclerosis on CVD events among HIV-infected and -uninfected adults in Botswana.

## Introduction

Human immune-deficiency virus (HIV) infection is associated with a 1.5–2 fold increase in risk for cardiovascular disease (CVD), even after controlling for traditional CVD risk factors [1–3]. Carotid intima media thickness (cIMT) is a commonly used surrogate marker of CVD among HIV-infected patients [4, 5], and has been variably increased in studies of HIV-infected individuals [5–7]. Presence of carotid plaque, conversely, has been consistently associated with HIV-infection [8, 9], and sometimes linked to exposure to protease inhibitors [5]. These associations have been largely studied in low HIV prevalence settings in North America and Europe versus the epicentre of the HIV epidemic in the sub-Saharan Africa region such as in Botswana [10].

The excess CVD risk among HIV-infected patients has been attributed to immune activation (monocyte activation, specifically), among other mechanisms [11–14]. One of the commonly-studied markers of monocyte activation in HIV-infected populations is soluble CD163 (sCD163) [15], a hemoglobin scavenger protein that is released in plasma by activated monocytes [16]. sCD163 is increased in HIV-infected patients [17–19], and has been consistently associated with the presence of non-calcified coronary plaque [20, 21], arterial inflammation [15], and all-cause mortality [22], but has not been consistently associated with increase in cIMT [23] nor CVD events [24] among HIV-infected patients. All of these studies have focused on HIV-infected patients in non-sub Saharan African settings.

Given concerns regarding a rising epidemic of non-communicable diseases (NCDs) among HIV-infected patients globally, our aim was to compare cIMT and carotid plaque between virally suppressed HIV-infected patients and HIV-uninfected controls in a high HIV-prevalence setting such as Botswana. Additionally, we sought to explore the association between traditional CVD risk factors, sCD163, and cIMT/plaque.

## Methods

### Study participants

We enrolled HIV-infected adults on ART and HIV-uninfected adult controls in Gaborone, Botswana, between February 2014 and April 2015. We balanced enrolment by gender and age (within 10-year age groups) among HIV-infected versus HIV-uninfected participants (but individual participants were not matched). HIV-infected participants were recruited from Princess Marina Hospital Infectious Disease Care Clinic (PMH-IDCC) in Gaborone. HIV-uninfected controls were recruited at a Gaborone Voluntary HIV testing centre. Study staff approached potentially eligible participants at these centers, and also enrolled eligible individuals who were referred to the study team by IDCC clinic or HIV testing centre staff.

Specific inclusion criteria for HIV-infected participants included documented HIV-1 RNA greater than 400 copies/ml and/or dual positive HIV enzyme linked immunosorbent assay (ELISA) prior to initiation of antiretroviral therapy (ART). Additionally, all HIV-infected participants had to have suppressed HIV-1 RNA (400 copies/ml or less) on all HIV-1 RNA measurements within 6 months of recruitment, and to have been on continuous ART for at least 12 months with no change in regimen within the 6 weeks preceding recruitment. Study participants had to be 30–50 years old (inclusive), and able to lie down for carotid ultrasound scanning. Pregnant women were excluded.

All participants provided written informed consent. The Botswana Ministry of Health Research & Development Committee, Princess Marina Hospital Ethics Committee and Partners Human Research Committee all approved the study.

### Study procedures

Data and samples were collected at one visit. Participants provided detailed medical history of stroke, myocardial infarction, diabetes mellitus, hypertension, chronic kidney disease, dyslipidaemia and cigarette smoking plus associated treatments where applicable. Family history of stroke and myocardial infarction was obtained for 1^st^ and 2^nd^ degree relatives.

HIV diagnosis method, ART history, associated CD4 count(s), and HIV-1 RNA level were obtained from participants`medical records. All participants underwent bilateral carotid ultrasound imaging and non-fasting blood samples were drawn for lipid profile, glycosylated haemoglobin (HBAIC) and soluble sCD163 testing.

### Carotid imaging

B-mode images at the distal (including bulb) 1-cm or more of the common carotid artery (CCA) were obtained with participant lying supine on an examination bed with the neck extended to reveal adequate views for imaging and head facing approximately 45 degrees to the right if the left CCA was being imaged and vice versa for imaging of the right CCA [25]. The study image acquisition protocol focused on ascertainment of cIMT at the distal 1-cm of the CCA while the rest of vessel structures were used as key anatomical landmarks (bulb, internal / external carotid arteries) for obtaining optimal images of the CCA and evaluate for evidence of plaque. Plaque was defined as any focal atherosclerotic lesion >1.5mm on a still image obtained at the beginning of the R-wave. The chosen cIMT measurement software (sonocalc) was also optimized to obtain cIMT measurement along the CCA as per manufacturer’s instructions. Still images during the beginning of the R-wave at the distal CCA were obtained in longitudinal view along lateral, anterior and posterior views on the left and right. All images were obtained using HFL38 13–6 MHz linear probe connected to Sonosite M-turbo ultrasound system (FUJIFILM Sonosite Inc, Bothell, WA, USA) during continuous electrocardiographic (ECG) monitoring. Two sonographers (MM & WM) were trained in this imaging technique and reading cIMTs by Dr Linda C. Hemphill (LCH) at Massachusetts General Hospital, Boston, USA. LCH provided on-going review of image acquisition and quality for all study subjects, and supervised subsequent reading of cIMTs using SonoCalc software (version 5.0 of 2011) in auto-mode. cIMT was measured at the distal 1cm of the far wall of the CCA and reviewer was blinded from participants’ clinical details. The overall cIMT measurement intra-reader bias on validation set of 20 adults was -0.00325mm with lower bound of -0.04694mm, upper bound of 0.040442mm for reader 1 and -0.02365mm with lower bound of -0.07651mm and upper bound of 0.029214mm for reader 2, while the inter-reader bias was 0.02435mm with lower bound of -0.04525mm and upper bound of 0.093948mm.

### Metabolic and monocyte activation laboratory testing

Total cholesterol (TC), high density cholesterol (HDL), direct low density cholesterol (LDL), and triglyceride levels were measured using calorimetric method while HBA1C was measured using turbidimitric immunoassay technique, both on COBAS Integra 400+ analyser (Roche Diagnostics, Mannheim, Germany). HIV-1 RNA level and CD4 counts obtained from the medical records had been measured using ultrasensitive HIV RNA real time PCR technique (Abbott analyser, Germany), and flow cytometry techniques on the BectonDickinson Biosciences FACS Calibre cytometer (Becton Dickinson, San Jose, California, USA), respectively. Soluble CD163 level was measured using enzyme linked immunosorbent assay (Trillium Diagnostics, Bangor, ME, USA) according to manufacturer’s instructions. All sCD163 results were above the lower limit of detection (LLOD) of the assay (minimum result was 71ng/ml for this study). Laboratory testing was performed at the Botswana-Harvard HIV Reference Laboratory, Gaborone, Botswana.

### Sample size/sower considerations and statistical analysis

Our study was powered to detect a minimum mean cIMT difference of 0.04 mm with 80% confidence if we enrolled a minimum of 175 HIV-infected participants and 175 HIV-uninfected controls. This difference in cIMT between HIV-infected versus HIV-uninfected controls was reported in a major systematic review [26] and in the largest report to date on the association between HIV and cIMT (the Fat Redistribution and Metabolic Changes in HIV infection or FRAM study [27]).

Baseline characteristics were summarized and compared between HIV-infected versus HIV-uninfected participants using Student`s t-test for continuous measures and Fisher`s exact test for categorical variables. For continuous measures, the Wilcoxon rank-sum test was performed as a sensitivity analysis; the conclusions of this non-parametric test matched those of the t-test hence results of the t-test are reported.

Univariate linear regression analysis models assessed the association between cIMT and the following variables: HIV status, HIV-related factors including pre-ART CD4 count (<350 vs. ≥350), CD4 nadir (<350 vs. ≥350), current CD4 count (<350 vs. ≥350), duration of HIV disease, ART regimen, duration of ART regimen; traditional CVD risk factors including age, sex, cigarette smoking, body mass index, waist circumference, waist-hip ratio, systolic blood pressure, diastolic blood pressure, mean arterial blood pressure, diagnosis of hypertension, use of anti-hypertensive treatment, HBA1C, non-HDL cholesterol, HDL cholesterol, LDL cholesterol, use of statin therapy; sCD163. The association between plaque and HIV status was assessed using log binomial model, testing prevalence of plaque among HIV-infected participants versus controls. The association between sCD163 and cIMT was explored by plotting sCD163 (y-axis) against cIMT (x-axis) and using a quadratic term. Interaction by HIV-status (interaction term) was assessed for each predictor variable included in univariate analysis. Predictor variables that were significant in univariate analysis with a cut-off point of p≤ 0.15 (in addition to avoiding collinear variables) were included in a stepwise linear regression model and variables were retained if p≤0.05. Both univariate and multivariate models were assessed for all participants, HIV-infected only and HIV-uninfected only.

## Results

### Demographics and clinical characteristics of HIV-infected and HIV-uninfected participants

Of the 432 participants enrolled, 208 (48%) were HIV-infected adults while 224 (52%) were HIV-uninfected controls. HIV-infected participants were older than HIV-uninfected controls (median age 39 versus 37 years, p<0.001). Females constituted 55% of the HIV-infected participants and 47% of HIV-uninfected controls respectively (Table 1).

**Table 1:** Demographics and clinical characteristics of HIV-infected patients and HIV-uninfected controls.

	HIV-infected Patients, n = 208 (48%)^a^	HIV-uninfected Controls, n = 224 (52%)^a^	P-value^b^
Age in years	39 (5)	37 (5)	<0.001
30–39	122 (43)	159 (57)	
40–50	86 (60)	65 (40)	
Female	114 (55%)	105 (47%)	0.10
*Cigarette Smoking*			
Ever	71 (34%)	57 (25%)	0.06
Current	15(7%)	32 (14%)	0.02
Pack Years	4.7 (6.7)	4.9 (6.4)	0.89
*Family History*			
Myocardial Infarction	2 (1%)	8 (4%)	0.11
Stroke	24 (12%)	35 (16%)	0.26
*Personal History*			
Diabetes Mellitus	1 (0%)	2 (1%)	1.0
Hypertension	36 (17%)	19 (8%)	0.009
Chronic Kidney Disease	7 (3%)	0 (0%)	0.006
Dyslipidaemia	17 (8%)	8 (4%)	0.06
*Medications*			
Thiazide Diuretic	35 (15%)	12 (5%)	<0.001
Calcium Channel Blocker	17 (8%)	5 (2%)	0.007
Beta-blocker	7 (3%)	3 (1%)	0.21
ACE inhibitor	5 (2%)	5 (2%)	1.0
HMG Co-A inhibitors	10 (5%)	1 (0%)	0.004
Fibrates	1 (0%)	0 (0%)	0.48
*Anthropometric Data*			
Systolic Blood Pressure (mmHg)	130.3 (15.7)	131.5 (14.4)	0.40
Diastolic Blood Pressure (mmHg)	85.1 (12.4)	84.8 (11.8)	0.78
Waist-hip ratio			
Females (≥0.85)	38 (49%)	37 (39%)	0.28
Males (≥0.90)	21 (33%)	21 (20%)	0.07
*CVD risk blood tests*			
Total cholesterol (mmol/L)	4.7 (1.1)	4.5 (1.1)	0.13
LDL-cholesterol (mmol/L)	2.9 (1.0)	2.8 (0.9)	0.33
HDL-cholesterol (mmol/L)	1.4 (0.5)	1.4 (0.4)	0.07
Triglycerides (mmol/L)	1.4(1.1)	1.1 (1.0)	0.005
Glycosylated haemoglobin (%)	5.3 (0.5)	5.6 (0.8)	<0.001
*HIV-parameters*		.	
HIV Disease Duration (years)	10 (3.2)	.	N/A
Duration on ART	8.6 (2.7)	.	N/A
CD4 nadir (cells/ul)	126(99)	.	N/A
*Proportion with CD4 nadir <350 (cells/ul)	141 (97)		
PreART CD4 count (cells/ul)	133(105)	.	N/A
Current CD4 count (cells/ul)	564 (231)	.	N/A
Proportion with undetectable VL	100%	.	N/A
Time since VL <400copies/ml (months)	3.1 (1.9)	.	N/A
Patients on NNRTI-based ART	155 (75%)	.	N/A
Patients on PI-containing ART	52 (25%)	.	N/A

Abbreviations: HIV, human immune deficiency virus; ACE, angiotensin converting enzyme; HMG-Co A, 3-hydroxy-3-methylglutaryl-coenzyme A; CVD, cardiovascular disease; LDL, low density lipoprotein; HDL, high density lipoprotein; CD4, cluster differentiation 4; VL, viral load; NNRTI, non-nucleoside reverse transcriptase inhibitor; PI, protease inhibitor

^a^ Means (standard deviations) for continuous measures; counts (percentages) for categorical measures

^b^ P-values are from t-test for continuous measures and from Fisher’s exact test for categorical measures.

*62 patients had missing nadir CD4 results

Prior cigarette smoking was reported by 34% of HIV-infected versus 25% of HIV-uninfected participants. However, significantly fewer HIV-infected participants reported current smoking (7%) compared with HIV-uninfected participants (14%, p = 0.02). Total cigarette pack years were similar in both groups. HIV-infected and -uninfected participants reported similar rates of family history of cerebrovascular or coronary artery events. HIV-infected participants were more likely to have a prior diagnosis of hypertension (17% versus 8%, p = 0.009) or chronic kidney disease (3% versus 0%, p = 0.006) than controls, respectively. There were no significant differences in rates of diabetes mellitus between study groups. Compared with HIV-uninfected controls, HIV-infected participants were more likely to be on thiazide diuretics (15% versus 5%, p<0.001) or calcium channel blockers (8% versus 2%, p = 0.007). While there was only a trend toward significance in dyslipidaemia between study groups (p = 0.06), HIV-infected participants were significantly more likely to be on statin therapy than HIV-uninfected controls (5% versus 0% respectively, p = 0.004). None of our study participants were on aspirin. Measured blood pressure and waist-hip ratios were not significantly different between study groups. Among the CVD risk blood tests, triglycerides levels were significantly higher but HBA1C was significantly lower among HIV-infected compared with HIV-uninfected participants (p = 0.005 and p<0.001, respectively).

Among HIV-infected participants, the median HIV disease duration was 10 years; the median time on ART was 9.3 years; and the median CD4 count at study enrolment was 536 cells/ul. Seventy-five percent of HIV-infected participants were taking non-nucleoside reverse transcriptase inhibitor (NNRTI)-based ART while the remaining 25% were on protease inhibitor (PI)-based ART. The median time since most recent viral load of ≤400 copies/ml was 3 months.

All participants in this cohort were free of clinical atherosclerotic cardiovascular disease except for 2 reported prior stroke and myocardial infarction (not shown).

### cIMT and monocyte activation, and carotid plaque among HIV-infected compared to HIV-uninfected participants

Overall, cIMT was symmetrically distributed with 4 outliers on the upper end of the cIMT distribution. No significant difference in cIMT was observed between HIV-infected participants (0.607 mm) vs. HIV-uninfected controls (0.599 mm, p = 0.37). Sensitivity analysis that excluded the four outliers did not alter this finding.

**Fig 1:**
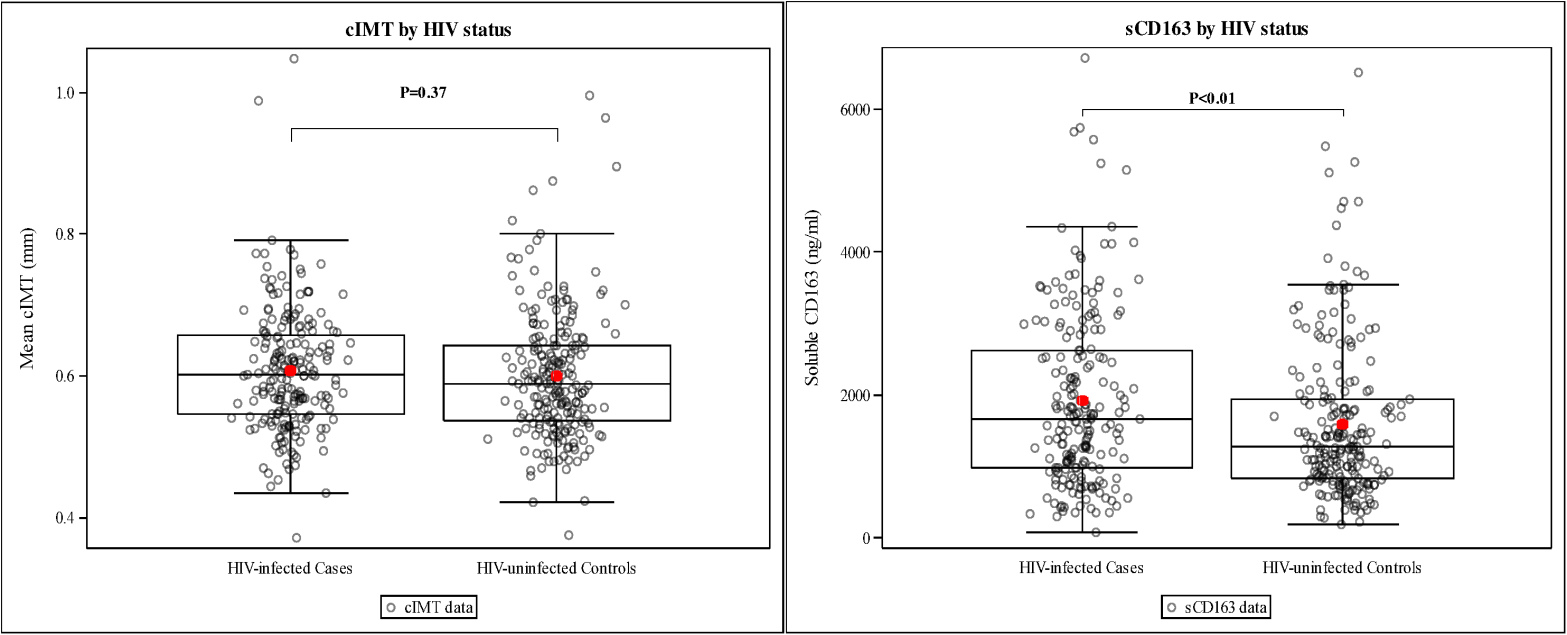
Carotid intima media thickness and sCD163 among HIV-infected patients and HIV-uninfected controls. The horizontal bars of the whiskers represent the 10^th^ and 90^th^ percentile while the upper and lower boundaries of the boxes represent the first and third quartiles. The dots on either side of the box-plot represent the lower and upper extremes of the data.

sCD163 was performed on samples from all 208 HIV-infected participants and on 223 out of 224 HIV-uninfected controls. HIV-infected participants had significantly higher sCD163 levels than HIV-uninfected controls, 1917± 1209 ng/ml versus 1593 ± 1080 ng/ml, p = 0.003 (Fig 1).

Plaque was present among 32 (15.4%) HIV-infected patients versus 16 (7.1%) HIV-uninfected patients. The unadjusted relative risk (95% CI) for having plaque among HIV-infected participants versus controls was 2.15 (1.22, 3.81; p = 0.008).

### Univariate analysis of CVD risk factors, monocyte activation and cIMT

Among all participants, several factors were significantly associated with increased cIMT in univariate analysis, as detailed in Table 2. Higher HDL cholesterol was associated with decreased cIMT. sCD163 was also not associated with cIMT in analyses of all participants or among HIV-infected participants (Table 2).

**Table 2:** Univariate predictors of cIMT, including traditional CVD risk factors, HIV factors and sCD163 among all study participants, HIV-infected participants, and HIV-uninfected participants.

	All participants		HIV-infected participants		HIV-uninfected participants	
Predictors Variable	Estimate (95% CI)	P-value	Estimate (95% CI)	P-value	Estimate (95% CI)	P-value
Age (per 10 year increase)	0.07 (0.06, 0.08)	<0.01	0.06 (0.04, 0.08)	<0.01	0.08 (0.06, 0.1)	<0.01
Sex (ref = male)	-0.01 (-0.027, 0.006)	0.23	-0.02 (-0.044, 0.003)	0.09	-0.002 (-0.026, 0.022)	0.85
*Personal History*						
Hypertension	0.053 (0.029, 0.078)	<0.01	0.045 (0.014, 0.075)	<0.01	0.066 (0.024,0.108)	<0.01
On Treatment for Hypertension	0.057 (0.032, 0.082)	<0.01	0.045 (0.014, 0.075)	<0.01	0.08 (0.035,0.125)	<0.01
*Anthropometric Data*						
Systolic Blood Pressure (per 10 mmHg)	0.02 (0.01, 0.02)	<0.01	0.02 (0.01, 0.03)	<0.01	0.01 (0.01, 0.02)	<0.01
Diastolic Blood Pressure (per 10 mmHg)	0.02 (0.01, 0.03)	<0.01	0.02 (0.01, 0.03)	<0.01	0.02 (0.01, 0.03)	<0.01
Mean Arterial Pressure (per 10 mmHg)	0.02 (0.01, 0.03)	<0.01	0.02 (0.01, 0.03)	<0.01	0.02 (0.01, 0.03)	<0.01
Body Mass Index (per 5 point increase)	0.012 (0.004, 0.019)	<0.01	0.02 (0.009, 0.032)	<0.01	0.007 (-0.003, 0.017)	0.19
Waist Circumference (per 10cm increase)	0.01 (0.01, 0.02)	<0.01	0.02 (0.01, 0.03)	<0.01	0.01 (0.00, 0.03)	0.05
Waist-hip ratio (per 0.5 increase)	0.16 (0.09, 0.23)	<0.01	0.20 (0.10, 0.30)	<0.01	0.14 (0.04, 0.24)	<0.01
*CVD risk blood tests*, *therapy*						
Non-HDL cholesterol (per 2 mmol/L)	0.04 (0.02,0.05)	<0.01	0.03 (0.01,0.05)	<0.01	0.04 (0.02,0.06)	<0.01
LDL-cholesterol (per 2 mmol/L)	0.04 (0.02,0.06)	<0.01	0.03 (0.01,0.05)	0.02	0.05 (0.02,0.07)	<0.01
HDL-cholesterol (per 2 mmol/L)	-0.04 (-0.08,0.00)	0.03	-0.06 (-0.11,-0.01)	0.02	-0.03 (-0.09,0.04)	0.42
Glycosylated haemoglobin (per 2% point)	0.03 (0.01,0.05)	0.01	0.00 (-0.04,0.05)	0.83	0.05 (0.02,0.08)	<0.01
Statin therapy	0.05 (-0.002, 0.103)	0.06	0.024 (-0.031, 0.079)	0.39	0.276 (0.101,0.451)	<0.01
*Monocyte Activation*						
sCD163 (per 1000ng/ml)	0.00 (0.00,0.01)	0.43	0.00 (-0.01,0.01)	0.72	0.00 (-0.01,0.01)	0.55
*HIV-factors*						
Duration of HIV disease (per 3 year increase)	N/A	N/A	0.01 (0.0, 0.02)	0.04	N/A	N/A
Duration of ART exposure (per 3 year increase)	N/A	N/A	0.01 (0.0, 0.03)	0.06	N/A	N/A
Current PI use (versus NNRTI use)	N/A	N/A	0.02 (-0.01, 0.04)	0.26	N/A	N/A
Nadir CD4 count (≥350 versus <350 cells/ul)	N/A	N/A	-0.08 (-0.15, -0.01)	0.02	N/A	N/A

Abbreviations: CVD, cardiovascular disease; HDL, high density lipoprotein; LDL, low density lipoprotein; sCD163, soluble CD163; HIV, human immune deficiency virus; ART, antiretroviral therapy; PI, protease inhibitor; NNRTI, non-nucleoside reverse transcriptase inhibitor.

Among both HIV-infected and HIV-uninfected participants in separate analyses, older age, history of hypertension, current use of anti-hypertensive medication, current systolic blood pressure, current diastolic blood pressure, current mean arterial pressure, waist circumference and waist-hip ratio were each associated with higher cIMT in univariate analyses. While sex did not reach a statistically significant association with cIMT in either group, the effect of sex was 10 fold higher on cIMT among HIV-infected versus controls. Body mass index, however, was associated with increased cIMT among HIV-infected but not HIV-uninfected participants.

In relation to lipid testing, higher non-HDL cholesterol and LDL cholesterol were also significantly associated with increased cIMT among both HIV-infected and HIV-uninfected participants. There was discordance in the association between increased cIMT for the other lipid indices and statin exposure: among HIV-infected participants neither HBA1C nor statin therapy was associated with cIMT however HDL cholesterol was inversely associated with increased cIMT. In contrast, among HIV-uninfected controls, an increase in HBA1C and current statin use was significantly associated with increased cIMT, but HDL cholesterol was not. HIV disease duration and nadir CD4 <350 cells/ul were associated with increased cIMT. ART duration trended toward significance (p = 0.06); type of ART (PI- versus NNRTI-based ART at time of enrolment) was not associated with cIMT (Table 2). sCD163 was not associated with cIMT in any group.

### Multivariate analysis of CVD risk factors and cIMT

After adjustment for age, waist-hip ratio, treatment for hypertension, HBA1C and non-HDL cholesterol, an association between HIV status and cIMT was not apparent (p = 0.22). Factors that remained significantly associated with increased cIMT in multivariate models including all participants were older age, higher non-HDL cholesterol, and being on treatment for hypertension (Table 3). Additionally, the significant associations between waist-hip ratio and HBA1C and cIMT that were observed in univariate modelling lost significance in the multivariate model (p = 0.21 and p = 0.66, respectively). Among only the HIV-infected participants, older age and higher waist-hip ratio remained significant in the final multivariate model (p = 0.02 and p = 0.02 respectively). Finally, among only HIV-uninfected participants, older age, HDL cholesterol and HBA1C were associated with higher cIMT (p<0.001, p = 0.05 and p = 0.01 respectively).

**Table 3:** Multivariate association between traditional CVD risk factors and cIMT among all participants and among HIV-infected participants.

	All participants		HIV-infected participants		HIV-uninfected	
Predictor Variable	Estimate (95% CI)	P-value	Estimate (95% CI)	P-value	Estimate (95% CI)	P-value
Age (per 5 year increase)	0.030 (0.023, 0.037)	<0.001	0.018 (0.003–0.032)	0.02	0.03579 (0.026–0.046)	<0.001
On treatment for hypertension	0.026 (0.003–0.050)	0.03	0.031 (-0.007–0.068)	0.11		
Non-HDL cholesterol	0.012 (0.005, 0.019)	<0.001			0.012 (0.003–0.022)	0.01
Waist-hip ratio			0.260 (0.050–0.471)	0.02		
HBA1C					0.013 (0.0001–0.027)	0.05

Abbreviations: HDL, high density lipoprotein; sCD163, soluble CD163; HBA1C, glycosylated haemoglobin

Greyed out areas indicate that these predictors were not significant in the final models

All of the final models above were examined after log transforming cIMT and were reassessed after excluding the four cIMT outliers; conclusions were similar and normality of error terms was not noticeably improved.

## Discussion

HIV-infected participants aged 30–50 years who have achieved viral suppression do not have higher cIMT when compared to HIV-uninfected controls in Botswana. Independent traditional CVD risk factors that were associated with cIMT differed for HIV-infected versus HIV-uninfected participants. sCD163 was elevated in HIV-infected participants versus controls but was not associated with presence of increased cIMT among all participants or among either of the HIV groups.

Our finding that cIMT among HIV-infected patients with well controlled disease was similar to cIMT in HIV-negative individuals has been observed in other studies, including in Ethiopia [28], Australia [29], and the US [8, 30]. In exploratory analyses that were unadjusted, we found that HIV-infection was associated with increased prevalence of carotid plaque, similar to findings from non-African HIV-infected persons [5]. The association between HIV infection and presence of carotid plaque merits further investigation because carotid plaque is a strong predictor of future clinical cardiovascular events (coronary artery disease, stroke) in the general population [31–34].

Furthermore, multiple established CVD factors associated with cIMT were associated with cIMT in univariate analyses in our study as has been reported by others [6, 7, 35–37]. Our final multivariate model highlighted the importance of the association between traditional CVD risk factors and cIMT. This same observation was made in a Brazilian cohort of 591 patients in whom traditional CVD risk factors predicted cIMT in multivariate analysis in those less than 40 years of age [6]. Findings of the Brazilian HIV cohort and the predominantly female HIV-infected clinical cohort in South Africa aligned with our findings and did not find an association between HIV-related factors and cIMT [7]. While other studies have reported the association between HIV disease duration and increase in cIMT, we did not find this association in adjusted analysis [38].

Taken together, prevalent reversible traditional CVD risk factors that predicted cIMT were well controlled among HIV-infected patients in our cohort, highlighting that it is feasible to control CVD risk factors among HIV-infected participants who are engaged in care when compared to asymptomatic controls in the same population. Despite Botswana primary care guidelines recommending aspirin for patients at increased risk for CVD such as those with known diabetes mellitus, none of our participants were on aspirin, probably due to low aspirin use among HIV infected patients even when it is indicated as has been well described by others [39, 40].

We observed that sCD163 was elevated in HIV even in the setting of viral suppression, a finding consistent with some studies [17, 18]. However, elevated sCD163 was not associated with cIMT or presence of plaque. On balance, a systematic review that also addressed the association between between sCD163 and cIMT among HIV-infected groups, revealed that sCD163 was not consistently associated with cIMT in non-African settings [23].

Other than age, the final CVD risk factors that independently predicted cIMT differed by HIV status. Receiving treatment for hypertension and waist-hip ratio predicted cIMT among HIV-infected participants while non-HDL cholesterol and HBA1C predicted cIMT among HIV-uninfected controls. With regard to effect of treatment of hypertension, our observation of higher prevalence of hypertension and treatment rates among HIV-infected than HIV-uninfected may indicate that hypertension treatment was a stronger surrogate marker of severe hypertension (and not just any elevated blood pressure for which treatment was not offered) among patients on care as compared to HIV-uninfected controls recruited at an HIV testing centre. We postulate that the reason waist-hip ratio was strongly associated with cIMT in HIV-infected adults (many of whom had been treated with older NRTIs) is likely due to induced excess visceral / central fat deposition [35, 41]. While non-HDL cholesterol predicted cIMT among HIV-uninfected patients but not HIV-infected participants in the final model, we suspect that higher rates of statin use among HIV-infected patients attenuated the effect of non-HDL cholesterol on cIMT [42, 43]. Finally, the apparent significant impact of HBA1C on cIMT among HIV-uninfected participants would be in keeping with HBA1C as a predictor of cIMT [44]. However, lack of this association among HIV-infected may reflect HBA1C as a less accurate measure of glucose intolerance in this population [45].

Our study was subject to several limitations. One of these was measurement of cIMT at the common carotid artery only, which is the most technically feasible site to image and ascertain presence of atherosclerosis using sonocal cIMT software. Evidence of early atherosclerosis occurs at the carotid bifurcation first, followed by proximal internal carotid artery, and any atherosclerosis seen in the common carotid artery often occurs late and therefore will likely be less common in younger patients [46]. Future studies in this setting evaluating cIMT as a surrogate marker of CVD risk among relatively younger HIV-infected patients should focus on evaluation of atherosclerosis at sites of initial CVD disease manifestation such as carotid bifurcation or the proximal internal carotid artery. Future work should also explore a broader array of markers of monocyte activation and assess whether monocyte activation is associated with endothelial injury and CVD events despite similar cIMT between HIV-infected and controls in Botswana. We did not enrol patients 50 years or older. We also did not assess and control for psychocosocial factors that may have contributed to the observed pre-clinical atherosclerosis in our study. Additional residual confounding that affected our study findings includes potential confounding by age when assessing association between ART duration / HIV disease duration and cIMT. Our relatively study small sample size precluded ability to assess factors associated with presence of carotid plaque. Finally, our cross-sectional design limited our ability to assess temporal associations between the various predictors and outcome of cIMT.

## Conclusion

In this study of virally suppressed individuals on ART in Botswana compared with demographically similar controls, HIV status was associated with persistent monocyte activation but was not associated with cIMT. While this increase in monocyte activation did not appear to translate into increased atherosclerosis in our participants, it may nevertheless have implications for future CVD events. Reassuringly, HIV-infected participants who were engaged in care in Gaborone were found to have good CVD risk management. This study represents the largest study to compare cIMT between virally suppressed and demographically similar controls, and to assess the association between sCD163 and cIMT and plaque in a sub-Saharan African setting. Future studies should enrol larger sample sizes so that they are adequately powered to robustly assess the relationship between HIV-status, carotid plaque and monocyte activation in sub-Saharan Africa.
